# Correlations between Personality Traits, Patient-Reported Outcome, and Chronic Prostatitis Symptoms in Men with Different Premature Ejaculation Syndromes

**DOI:** 10.1155/2022/8049976

**Published:** 2022-04-08

**Authors:** Jingjing Gao, Rui Gao, Xi Liu, Chengfu Li, Pan Gao, Junhua Du, Xiansheng Zhang

**Affiliations:** ^1^Department of Urology and Andrology, The First Affiliated Hospital of Anhui Medical University, Anhui Province, China; ^2^Department of Urology and Andrology, The Second People's Hospital of Fuyang City, Fuyang City, Anhui Province, China

## Abstract

Although the personality traits (PT), patient-reported outcome (PRO), and chronic prostatitis (CP) symptoms in premature ejaculation (PE) have been evaluated, there was no study to assess their correlations in men with different PE syndromes. The purpose of this study was to assess the correlations between the PT, PRO, and CP symptoms in men with different PE syndromes. Between January 2019 and January 2021, a cross-sectional field study was conducted in our andrology clinic. Men with the complaints of PE were divided into lifelong PE (LPE), acquired PE (APE), variable PE (VPE), and subjective PE (SPE). All subjects were required to complete a verbal questionnaire with the PRO, National Institutes of Health Chronic Prostatitis Symptom Index (NIH-CPSI), and Temperament and Character Inventory (TCI-R). Finally, 479 men with the complaints of PE and 365 without the complaints of PE were enrolled. The incidence of PE syndromes in PE complaint group was as follows: LPE 16.70%, APE 48.85%, VPE 11.27%, and SPE 23.17%. Mean ages in PE complaint group were 42.53 ± 12.25 years. In the PE complaint group, the novelty seeking (NS) scores were strongest correlated with the *personal distress* and *quality of life (QOL)*. The harm avoidance (HA) scores were strongest correlated with the *severity of PE* and *pain syndromes*. The self-transcendence (ST) scores were strongest correlated with the *satisfaction with sexual intercours*e and *QOL*. In addition, strongest association between the total scores of NIH-CPSI and the NS or ST scores was also found in the APE group. The HA scores were also strongest correlated with the total scores of NIH-CPSI in SPE. Strongest association between the total scores of NIH-CPSI and the NS/TI or ST/CI scores was also found in the APE group. The HA/TI scores were also strongest correlated with the total scores of NIH-CPSI in SPE.

## 1. Introduction

Premature ejaculation (PE) is one of the most common types of ejaculatory dysfunction, with a high prevalence. According to the guidelines from the International Society for Sexual medicine (ISSM)[1, 2], the diagnostic criteria for PE should include three parts: (1) short intravaginal ejaculation latency time (IELT), (2) reduction in ejaculatory control, and (3) negative sexual consequences in the patient and in the partner, e.g., anxiety, depression, or decreased sexual desire.

Although these parts played the important roles in the diagnosis of PE, the definition did not cover all aspects. For example, some men complained of PE but with the normal or extended IELTs. In addition, their decreased sexual satisfaction has affected the harmonious relationship between couples. To solve these clinical issues, Waldinger and Schweitzer have proposed a new classification of PE. Besides the lifelong PE (LPE) and acquired PE (APE), two additional PE subtypes (subjective PE (SPE) and variable PE (VPE)) have been added [3, 4].

From the data from our previous study (including 1988 outpatients with PE complaints), the prevalences of four PE syndromes in outpatients were 35.66% (709/1988) for LPE, 28.07% (558/1988) for APE, 12.73% (253/1988) for VPE, and 23.54% (468/1988) for SPE [5]. However, in another study in male population of Anhui province (a total of 3016 men evaluated) [6], we found that the distribution of the four PE syndromes in men with the complaints of PE was in the order of VPE (343/778, 44.09%), SPE (193/778, 24.81%), APE (146/778, 18.77%), and LPE (96/778, 12.34%).

The patient-reported outcome (PRO) measures and National Institutes of Health Chronic Prostatitis Symptom Index (NIH-CPSI), which were used to assess PE and chronic prostatitis (CP) symptoms, have been widely used in previous studies [7–10]. The PRO starts from many aspects, including perception of ejaculatory control, satisfaction with ejaculatory control, and sexual intercourse. The NIH-CPSI included the *pain*, *urinary symptoms*, and *quality of life (QOL).* The results from our previous study showed that men with PE (IELT < 1 min) were more likely to report worse PRO scores (*reduced ejaculatory control*: 0.88 ± 0.74 vs. 2.47 ± 1.12; *sexual satisfaction*: 0.89 ± 0.76 vs. 2.72 ± 1.01; *increased personal distress*: 2.83 ± 0.96 vs. 1.34 ± 0.98; and *interpersonal difficulty*: 2.31 ± 1.24 vs. 1.27 ± 1.00) than men without PE [9]. Another study showed that men with the complaints of PE might reported worse NIH-CPSI scores, and total and subdomain of NIH-CPSI scores were higher in men with APE [10].

Personality was found to associate with PE [11, 12]. As the internal organization of affective, emotional, cognitive, and conceptual systems, personality could determine human unique repose to the environment. Temperament and Character Inventory (TCI) scale is widely used to measure personality [13–15]. It has provided a comprehensive biopsychosocial model of personality and divided personality into seven dimensions that vary widely in the general population [16]. The results from our recent study have investigated the temperament-character traits (evaluated by TCI-Revised) and attitudes toward PE. We found that men with the complaints of PE reported lower novelty seeking (NS) and self-transcendence (ST) scores and higher harm avoidance (HA) than men without complaints of PE. In addition, men with VPE have shown the highest HA scores and lowest NS scores than men with other types of PE [12].

Although the personality and CP symptoms might play important roles in precipitating or maintaining PE, there was no study to assess their association in men with the complaints of PE, especially in men with four PE syndromes. Therefore, based on the new definition of PE [3, 4], we investigated the above issues with the PRO, NIH-CPSI, and TCI-R.

## 2. Materials and Methods

### 2.1. Subjects Enrolled

Between January 2019 and January 2021, an observational and cross-sectional field study was conducted in the First Affiliated Hospital of Anhui Medical University. From the beginning of study, our team gradually established a database of all subjects. All men who have complaints of PE were enrolled from the andrology clinic of our hospital. In addition, other men with no PE complaints were enrolled from the health examination center of our hospital.

Before enrollment, the medical and sexual histories of all subjects were carefully evaluated by an andrology doctor. The inclusion criteria were as follows: (a) age of men ≥ 18 years; (b) in a heterosexual, stable, and monogamous sexual relationship with the same female partner ≥ 6 months; and (c) men can comprehend and speak Chinese. Men on medications whose ejaculatory function could be affected were excluded (e.g., selective serotonin reuptake inhibitors).

### 2.2. Study Design

This study was reviewed and approved by the Anhui Medical University Research Subject Review Board. Because of some subjective questions in the study, a prestudy (*n* = 30) was conducted to modify the questionnaire to ensure it is comprehensive and easily understandable. In addition, subjects were informed about the survey and those who participated were asked to provide written consent.

With a face-to-face interview, all subjects were required to complete a verbal questionnaire (including demographic information (e.g., *age*, *body mass index* (BMI)*, lifestyle*, *educational and occupational status*, and *resident*), sexual history [e.g., *frequency of sexual intercourse*, *duration of PE complaints*, and *self-estimated IELT*), medical history and comorbidities, the Chinese version of TCI-R, PRO measures, and NIH-CPSI). Cronbach's alpha for the TCI-R, PRO measures, and NIH-CPSI in our study were 0.79, 0.81, and 0.77, respectively.

### 2.3. Definition of PE

**Figure 1:**
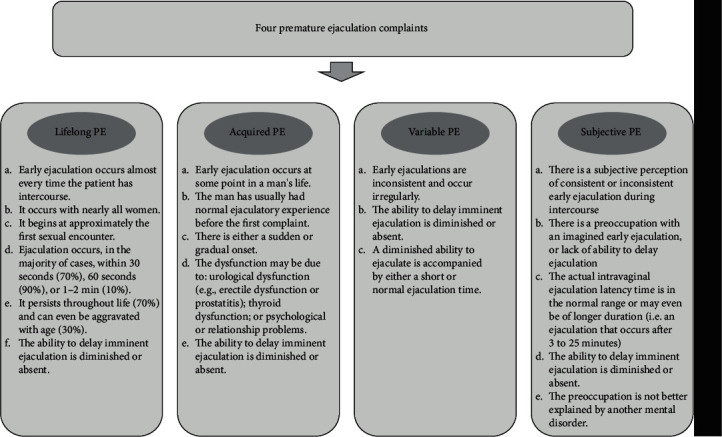
Classification and definition of premature ejaculation.

- Men with the complaints of PE: men who dissatisfied with their time to ejaculation
- Men with four PE syndromes: according to the new classification of PE (Figure 1), proposed by Waldinger and Schweitzer [3, 4], men with the complaints of PE were diagnosed with LPE, APE, VPE, or SPE

### 2.4. TCI-R

The Chinese version of TCI-R is a version of the TCI series scale which is widely used for assessing personality of adult population in China [12]. It has 240 items (each item rated on 5-level scale from completely inconsistent (*score: 1*) to completely in line (*score: 5*)) and consists of 4 dimensions of temperament-inventory (TI) (novelty seeking (NS), harm avoidance (HA), reward dependence (RD), and persistence (PS)) and 3 dimensions of character inventory (CI) (self-directedness (SD), cooperativeness (CO), and self-transcendence (ST)). The scale is suitable for people with different cultural backgrounds and has been reported good reliability and validity in our previous study [12].

### 2.5. PRO

**Figure 2:**
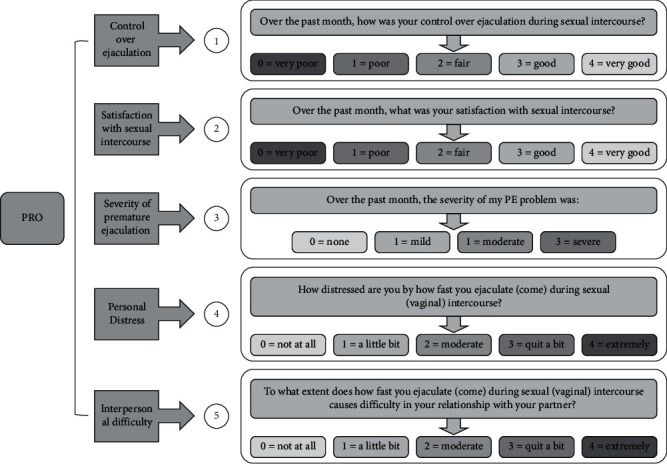
The Chinese version of PRO questionnaire.

The Chinese version of PRO questionnaire contains five measures and has been used in our previous study [9], including control over ejaculation (response scale ranging from very poor (*score 0*) to very good (*score 4*)), satisfaction with sexual intercourse (response scale ranging from very poor (*score 0*) to very good (*score 4*)), severity of PE (response scale ranging from none (*score 0*) to severe (*score 4*)), personal distress (response scale ranging from not at all (*score 0*) to extremely (*score 4*)), and interpersonal difficulty (response scale ranging from not at all (*score 0*) to extremely (*score 4*)). Detailed introduction of PRO questionnaire is shown in Figure 2.

### 2.6. NIH-CPSI

The Chinese version of NIH-CPSI is a reliable, convenient, self-administered index. It has been widely used to assess the severity of CP syndromes in China [10, 17, 18]. The questionnaire of NIH-CPSI has 9 items and consists of the measures of pain symptoms (total of items 1–4), urinary symptoms (total of items 5-6), and QOL (total of items 7–9). In addition, based on the total of items 1 to 6, the severity of pain and urinary symptoms in PE complaints has also been evaluated and classified as mild (10-14 points), moderate (15-29 points), or severe (>30 points).

### 2.7. Statistical Analysis

All data were analyzed by the SPSS software (SPSS Inc., Chicago, IL, USA) version 13.0. Data are expressed as mean ± standard deviation or number (percentage), as appropriate. Descriptive statistics were used to summarize the demographic information and presence of comorbidities in men with the complaints of PE and four PE syndromes.

Differences between men in PE and no PE complaint groups were assessed by the dependent *t*-test or chi-square test, as appropriate. Differences among four PE syndromes were assessed by one-way analysis of variance or chi-square test, as appropriate. Differences between two PE syndromes were assessed by *SNK*-q test.

Because the ages of men with PE complaints ranged from 20 to 66 years, and PE have been found to be associated with age, correlations between the outcomes of PRO measures, NIH-CPSI, and TCI-R in the PE complaint group were assessed by partial correlations (adjusted for age). For all of the tests, *P* < 0.05 was deemed statistically significant.

## 3. Results

### 3.1. Demographic Information

Finally, of 1104 men who met the inclusion criteria, 844 men (including 479 (479/844; 56.75%) men with the complaints of PE and 365 (365/844; 43.25%) men without the complaints of PE) were enrolled and finished the study, with a response rate of 76.45% (distribution of reasons for men discontinued the study: “incomplete information” (65/1104, 5.89%), “withdrawal of consent” (105/1104, 9.51%), and “other reasons” (90/1104, 8.15%)).

Based on the new classification, the incidence of four PE syndromes in men with PE complaints was as follows: LPE 16.70% (80/479), APE 48.85% (234/479), VPE 11.27% (54/479), and SPE 23.17% (111/479). Mean ages, BMI scores, and self-estimated IELTs in PE complaint group were 42.53 ± 12.25 years, 25.12 ± 4.02 kg/m^2^, and 2.23 ± 1.29 minutes, whereas those in no PE complaint group were 37.72 ± 9.02 years, 24.04 ± 3.31 kg/m^2^, and 3.65 ± 1.82 minutes, respectively. Detailed demographic information is shown in Table 1.

### 3.2. Outcomes of PRO, NIH-CPSI, and TCI-R in Men with the Complaints of PE

From Table 2, significant differences were found between men with and without PE complaints, in terms of men responses to PRO (all subdomain except for *severity of PE*) and NIH-CPSI (including total and all subdomain scores) (*P* < 0.001 for all).

Compared with men in no PE complaint group, men in PE complaint group have reported lower scores of *control over ejaculation* (1.01 ± 0.82 vs. 2.56 ± 1.15) and *satisfaction with sexual intercourse* (0.85 ± 0.75 vs. 2.80 ± 1.21) and higher scores of *personal distress* (2.63 ± 0.93 vs. 1.27 ± 0.86) and *interpersonal difficulty* (2.35 ± 1.54 vs. 1.20 ± 0.95).

For the total and subdomain (including *pain*, *urinary symptoms*, and *QOL*) scores of NIH-CPSI, their mean scores in men with PE complaint group were all significantly higher than those in men without PE complaint group (*P* < 0.001 for all). Mean *total scores* of NIH-CPSI in men with and without the complaints of PE were 23.12 ± 5.42 and 12.92 ± 3.50, respectively. Based on the classification of *severity of pain and urinary symptoms*, the percentage of mid, moderate, and severe of *pain and urinary symptom*s in PE complaint group were 37.79% (181/479), 32.99% (158/479), and 29.23% (140/479), respectively.

**Figure 3:**
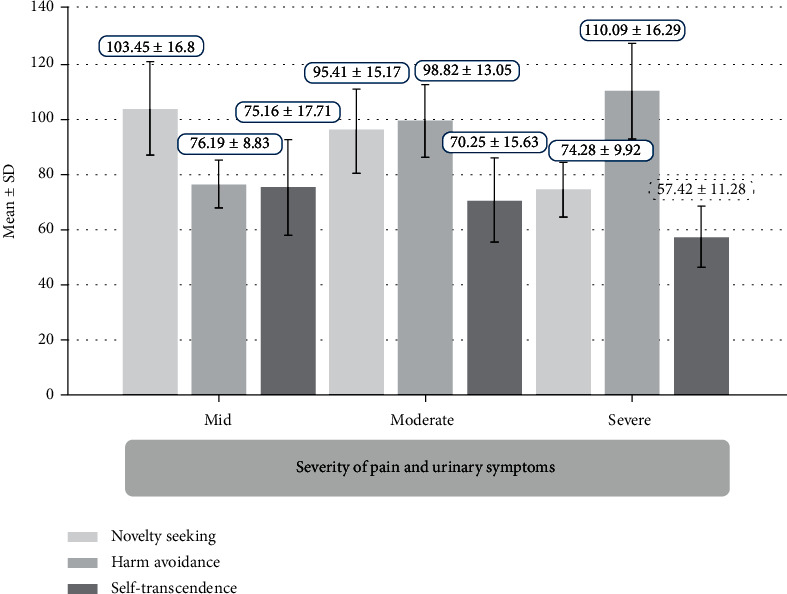
Mean scores of NS/TI, HA/TI, and ST/CI in different severity of pain and urinary symptoms.

In addition, the NS/TI, HA/TI, and SD/ST scores in men with the complaints of PE were significantly different than those in men without the complaints of PE (*P* < 0.001 for all). Men with the complaints of PE might reported lower scores of NS/TI (92.01 ± 12.03 vs. 96.53 ± 10.21) and ST/CI (68.40 ± 15.23 vs. 72.43 ± 16.69) and higher scores of HA/TI (97.00 ± 12.44 vs. 92.04 ± 11.27). Mean scores of NS/TI, HA/TI, and ST/CI in different severity of pain and urinary symptoms are shown in Figure 3.

### 3.3. Outcomes of PRO, NIH-CPSI, and TCI-R in Men with Four PE Syndromes

Similarly, significant differences were also found among four PE syndromes with respect to the outcomes of PRO, NIH-CPSI, and TCI-R (except for RD/TI, PS/TI, SD/CI, and CO/CI) (*severity of PE*: *P* = 0.032; *interpersonal difficulty*: *P* = 0.024; and others: *P* < 0.001).

Detailed outcomes of PRO, NIH-CPSI, and TCI-R in men with four PE syndromes are shown in Table 2.

### 3.4. Correlations between the Outcomes of PRO, NIH-CPSI, and TCI-R in Men with the Complaints of PE

After adjusted for ages, the correlations between the outcomes of PRO, NIH-CPSI, and TCI-R in men with the complaints of PE were evaluated (Table 3).

For the outcomes of PRO, the NS/TI scores in men with the complaints of PE were strongest negatively correlated with the subdomain of *personal distress* (Adjust *r* = −0.62, *P* < 0.001). Their HA/TI scores were strongest positively correlated with the subdomain of *severity of PE* (Adjust *r* = 0.68, *P* < 0.001). Their ST/CI scores were positively strongest correlated with the subdomain of *satisfaction with sexual intercourse* (Adjust *r* = −0.70, *P* < 0.001).

For the outcomes of NIH-CPSI, the NS/TI scores in men with the complaints of PE were strongest negatively correlated with the subdomain of *QOL* (Adjust *r* = −0.64, *P* < 0.001). Their HA/TI scores were strongest positively correlated with the subdomain of *urinary syndromes* (Adjust *r* = 0.66, *P* < 0.001). Their ST/CI scores were negatively strongest correlated with the subdomain of *QOL* (Adjust *r* = −0.69, *P* < 0.001).

### 3.5. Correlations between the Outcomes of PRO, NIH-CPSI, and TCI-R in Men with Different PE Syndromes

Furthermore, the correlations between the total scores of NIH-CPSI and NS/TI, HA/TI, or ST/CI in men with different PE syndromes were also assessed (Table 4).

The NS/TI scores were strongest negatively correlated with the total scores of NIH-CPSI in men with APE (Adjust *r* = −0.70, *P* < 0.001). Similarly, strongest negative correlations in APE were also found between the ST/CI scores and the total scores of NIH-CPSI (Adjust *r* = −0.65, *P* < 0.001). In addition, the HA/TI scores were strongest positively correlated with the total scores of NIH-CPSI in SPE (Adjust *r* = 0.68, *P* < 0.001).

## 4. Discussion

Sexual psychology is the psychological state and processes related to sexual desire and sexual behavior on the basis of sexual physiology [19, 20]. Temperament and character, as the important composition of personality, might be involved in the pathogenesis of sexual dysfunction through influencing the state of sexual psychology [11, 12, 21–23].

From previous psychiatric researches, temperament and character were often evaluated by using TCI-R questionnaires [24–26]. They were derived from Cloninger's personality model and contained four dimensions about temperament and three dimensions about character. Temperament was an automated reaction of experience and often recognized as related to biological genetics. It has the characteristic of lifetime stable and moderate hereditary. In addition, character was considered to be related to environmental factors. It was gradually mature through individual learning and social experience.

PE was the most common males' sexual dysfunction and had more complex etiology, including the internal and external factors. PE was also considered to associate with the subjective and objective factors. For example, the etiology of LPE might be associated with the sensitivity of the penile nerve, but the etiology of VPE might be related to the negative psychological burden [6, 10, 27].

In the past, there were few studies to assess the effects of temperament and character on PE and its associated factors. This is the first study to evaluate the effects of temperament and character on men with the complaints of PE/different PE syndromes (assessing by PRO) and their CP symptoms (assessing by NIH-CPSI). Findings from this study might play an important role in revealing the pathogenesis of PE.

The results from this study showed that men with lower scores of NS/TI reported worse personal distress in the PE complaint group. In addition, the worse severity of PE was observed when men with the complaints of PE reported higher HA/TI scores. In the meantime, the positive relationships between the ST/CI scores and sexual satisfaction were also found in PE complaint group.

Previous study confirmed the above findings. With the index of PE, strongest relationships between the NS/TI scores and distress of PE were found in men with the complaints of PE. These patients were more likely to reported worse sexual satisfaction with the decreased ST/CI scores [12]. In addition, another study conducted by Kempeneers et al. has shown that men combining generalized and lifelong PE with IELT less than 30 s reported lower sexual satisfaction and higher distress and HA/TI scores [11].

The NS/TI is defined as the activation of behavior, including the impulsive response to novel stimulation and the tendency to avoid setbacks. The HA/TI reflect the inhibition of emotion and behavior when we faced harmful stimulation. The ST/CI is a characteristic related to mental state that individuals regard subject and object as a unity and considered them to be interdependent to each other. According to the findings between temperament/character and PRO outcomes in men with PE, we speculated the following circles: Temperament and character might affect patients' psychological and behavior states. For example, patients who reported lower NS/TI and higher HA/TI scores were more likely to have negative feelings (e.g., anxiety, depression) and behaviors (e.g., avoidance of sexual intercourse and not willing to seek treatment). These negative effects would further aggravate the severity of PE and influence the sexual relationships between patients and their partners.

Chronic prostatitis, a risk factor of premature ejaculation, has a greater incidence of comorbid disease [28]. Screponi et al. found that 56.5% of patients had prostatic inflammation and 47.8% had chronic bacterial prostatitis, according to a study of premature ejaculation patients [29]. CP symptoms in men with the complaints of PE were evaluated by the NIH-CPSI, which contained three dimensions of pain, urinary symptoms, and QOL. In our study, NIH-CPSI was firstly used to assess its relationships with the personality traits (PT) in men with different PE syndromes. In the PE complaint group, findings showed that the NS/TI and ST/CI often influenced the QOL scores of NIH-CPSI, and the HA/TI were more likely associated with the urinary symptoms scores of NIH-CPSI. Because the NS/TI and ST/CI were related to the activation of behavior and mental state, respectively, lower NS/TI and ST/CI scores were associated with the decline of QOL through the decreased behavioral activity and psychological burden.

Additionally, because CP symptoms and psychological factors were considered the important roles in the etiology of APE and SPE, respectively, the NS/TI and HA/TI scores were found to associate with the total scores of NIH-CPSI in APE group, and the ST/CI scores were associated with those in SPE group.

However, several limitations should be considered. First, generalizability of this study may be limited by the fact that it was conducted in a single cultural/societal background. In addition, there were few studies on the effects of temperament and character on PE in the general male population. Second, patients might feel obliged to give socially acceptable answers when they answered some private, sensitive, and subjective questions with the face-to-face interview.

## 5. Conclusion

This is the first study to evaluate the correlations between PT (assessing by TCI-R) and PRO or CP symptoms (assessing by NIH-CPSI) in men with different PE syndromes in China.

In the PE complaint group, the NS/TI scores were strongest correlated with the *personal distress* and *QOL*. The HA/TI scores were strongest correlated with the *severity of PE* and *urinary syndromes*. The ST/CI scores were strongest correlated with the *satisfaction with sexual intercours*e and *QOL*. Strongest association between the total scores of NIH-CPSI and the NS/TI or ST/CI scores was also found in the APE group. The HA/TI scores were also strongest correlated with the total scores of NIH-CPSI in SPE.

Above the preliminary conclusions, further researches were needed to confirm. In addition, as an early classification method, the four types of PE has certain limitations and should be improved in the later research.

## Figures and Tables

**Table 1:** Demographic information and presence of comorbidities in men with different PE syndromes.

Factors	Total	PE complaint	No PE complaint	*P ^A^* value	Four PE syndromes				*P ^B^* value
					LPE	APE	VPE	SPE	
*N* (%)	844	479 (56.75%)	365 (43.25%)	NA	80 (16.70%)	234 (48.85%)	54 (11.27%)	111 (23.17%)	NA
Age (years)	40.45 ± 10.83	42.53 ± 12.25	37.72 ± 9.02	<0.001	36.69 ± 9.97^a,b,c^	49.49 ± 12.02^a,c,d^	30.51 ± 7.75^a,b,d^	37.93 ± 9.21^a,b,c^	<0.001
BMI (kg/m^2^)	24.65 ± 3.85	25.12 ± 4.02	24.04 ± 3.31	<0.001	21.76 ± 4.02^b,c,d^	26.59 ± 3.90^a,c,d^	25.35 ± 4.11^a^	24.34 ± 3.45^a,b^	0.55
Frequency of sexual intercourse (times/four weeks)	5.73 ± 2.90	5.41 ± 2.94	6.15 ± 2.30	<0.001	4.45 ± 2.51^b,c^	5.82 ± 2.72^a,c,d^	6.16 ± 3.05^a,b,d^	4.88 ± 2.88^a,b,c^	<0.001
Duration of PE complaint (months)	2.31 ± 1.55	2.31 ± 1.55	NA	NA	0.82 ± 0.30^b,c,d^	3.35 ± 1.27^a,c,d^	1.27 ± 1.01^a,b,d^	1.69 ± 0.74^a,b,c^	<0.001
Self-estimated IELT (minutes)	2.85 ± 1.54	2.23 ± 1.29	3.65 ± 1.82	<0.001	1.10 ± 0.63^b,c,d^	1.86 ± 0.94^a,c,d^	2.83 ± 1.52^a,b,d^	3.55 ± 1.42^a,b,c^	<0.001
Smoking, *n* (%)	511 (60.55%)	312 (65.14%)	199 (54.52%)	0.002	43 (53.75%)	189 (80.77%)	28 (51.85%)	52 (46.85)	<0.001
Exercise, *n* (%)	403 (47.75%)	201 (41.96%)	202 (55.34%)	<0.001	32 (40.00%)	79 (33.76%)	29 (53.70%)	61 (54.95%)	<0.001
Educational status, *n* (%)				0.118					<0.001
High school or less	267 (31.64%)	162 (33.82%)	105 (28.77%)		22 (27.50%)	78 (33.33%)	22 (40.74%)	40 (36.04%)	
Higher education	577 (68.36%)	317 (66.18%)	260 (71.23%)		58 (72.50%)	156 (66.67%)	32 (59.26%)	71 (63.96%)	
Occupational status, *n* (%)				0.334					<0.001
Student	213 (25.24%)	130 (27.14%)	83 (22.74%)		16 (20.00%)	70 (29.91%)	12 (22.22%)	32 (28.83%)	
Employed	456 (54.03%)	252 (52.61%)	204 (55.89%)		49 (61.25)	118 (50.43%)	29 (53.70%)	56 (50.45%)	
Unemployed	176 (20.85%)	97 (20.25%)	79 (21.64%)		15 (18.75%)	46 (19.66%)	13 (24.07%)	23 (20.72%)	
Resident, *n* (%)				0.345					<0.001
Urban	389 (46.09%)	214 (44.68%)	175 (47.95%)		33 (41.25%)	104 (44.44%)	22 (40.74%)	55 (49.55%)	
Rural	455 (53.91%)	265 (55.32%)	190 (52.05%)		47 (58.75%)	130 (55.56%)	32 (59.26%)	56 (50.45%)	

PE = premature ejaculation; LPE = lifelong PE; APE = acquired PE; VPE = variable PE; SPE = subjective PE; NA = not applicable. Data are expressed as mean ± standard deviation or number (percentage), as appropriate. *P^A^*: differences between men in PE and no PE complaint groups were assessed by the dependent *t*-test or chi-square test, as appropriate. *P^B^*: differences among four PE syndromes were assessed by one-way analysis of variance or chi-square test, as appropriate. Differences between two PE syndromes were assessed by *SNK*-q test. ^a^Significant difference compared with LPE. ^b^Significant difference compared with APE. ^c^Significant difference compared with VPE. ^d^Significant difference compared with SPE.

**Table 2:** Outcomes of the PRO, NIH-CPSI, and TCI-R questions in men with difference PE syndromes.

Factors	Total	PE complaint	No PE complaint	*P ^A^* value	Four PE syndromes				***P*** ^***B***^ **value**
					LPE	APE	VPE	SPE	
PRO questions, scores									
Control over ejaculation	1.68 ± 0.96	1.01 ± 0.82	2.56 ± 1.15	<0.001	0.90 ± 0.72^b,c,d^	0.86 ± 0.65^a,c,d^	1.45 ± 0.88^a,b,d^	1.17 ± 0.90^a,b,c^	<0.001
Satisfaction with sexual intercourse	1.69 ± 1.01	0.85 ± 0.75	2.8 ± 1.21	<0.001	0.82 ± 0.64^c,d^	0.80 ± 0.71^c,d^	0.92 ± 0.75^a,b^	0.96 ± 0.68^a,b,c^	<0.001
Severity of PE	2.26 ± 0.71	2.26 ± 0.89	NA	NA	2.29 ± 0.85^b,c,d^	2.36 ± 1.13^a,c,d^	2.05 ± 1.06^a,b,d^	2.13 ± 0.79^a,b,c^	0.032
Personal distress	2.04 ± 0.87	2.63 ± 0.93	1.27 ± 0.86	<0.001	2.42 ± 0.67^b,d^	2.65 ± 0.95^a,c,d^	2.40 ± 0.65^b,d^	2.84 ± 0.86^a,b,c^	<0.001
Interpersonal difficulty	1.85 ± 1.32	2.35 ± 1.54	1.20 ± 0.95	<0.001	2.24 ± 1.32^b,c,d^	2.30 ± 1.42^a,d^	2.29 ± 1.40^d^	2.57 ± 1.38^a,b,c^	0.024
NIH-CPSI question, scores									
Total score	18.71 ± 4.21	23.12 ± 5.42	12.92 ± 3.50	<0.001	16.72 ± 4.69^b,c,d^	29.38 ± 5.73^a,c,d^	13.66 ± 3.15^a,b,d^	19.14 ± 4.66^a,b,c^	<0.001
Pain score	10.18 ± 3.46	12.67 ± 5.85	6.92 ± 2.77	<0.001	7.52 ± 5.46^b,c,d^	16.11 ± 4.92^a,c,d^	7.54 ± 2.77^a,b,d^	11.64 ± 3.32^a,b,c^	<0.001
Urinary symptoms score	3.52 ± 1.75	4.49 ± 2.02	2.24 ± 1.28	<0.001	4.23 ± 1.81^b,c,d^	5.61 ± 2.40^a,c,d^	2.43 ± 1.35^a,b,d^	3.31 ± 1.43^a,b,c^	<0.001
Quality of life impact score	5.01 ± 2.03	5.96 ± 2.42	3.76 ± 1.52	<0.001	4.97 ± 2.34^b,c,d^	7.66 ± 3.56^a,c,d^	3.69 ± 1.28^a,b,d^	4.19 ± 2.68^a,b,c^	<0.001
TCI-R question: temperament inventory, scores									
Novelty seeking	93.97 ± 11.34	92.01 ± 12.03	96.53 ± 10.21	<0.001	90.28 ± 9.73^b,c,d^	93.35 ± 11.08^a,c,d^	91.12 ± 10.45^a,b,d^	90.88 ± 8.83^a,b,c^	<0.001
Harm avoidance	94.85 ± 11.57	97.00 ± 12.44	92.04 ± 11.27	<0.001	98.02 ± 13.29^b,c,d^	97.21 ± 11.59^a,c,d^	99.78 ± 14.07^a,b,d^	94.45 ± 10.29^a,b,c^	<0.001
Reward dependence	94.77 ± 10.92	94.58 ± 10.82	95.02 ± 11.33	0.475	94.90 ± 10.80	93.82 ± 10.65	95.43 ± 11.26	95.52 ± 10.84	0.462
Persistence	126.42 ± 13.88	127.13 ± 13.68	125.49 ± 14.59	0.483	129.20 ± 14.62	125.83 ± 12.09	127.56 ± 13.30	128.19 ± 14.67	0.454
TCI-R question: character inventory, scores									
Self-directedness	134.26 ± 18.05	135.07 ± 18.85	133.20 ± 17.42	0.415	134.35 ± 19.01	135.27 ± 18.54	135.62 ± 19.27	134.89 ± 18.16	0.582
Cooperativeness	128.62 ± 17.80	129.15 ± 18.04	127.92 ± 17.71	0.665	128.52 ± 18.26	129.76 ± 18.03	127.15 ± 17.39	129.30 ± 19.32	0.620
Self-transcendence	70.15 ± 15.67	68.40 ± 15.23	72.43 ± 16.69	<0.001	67.08 ± 13.72^b,c,d^	70.03 ± 16.21^a,b,d^	67.71 ± 15.86^a,b,d^	66.27 ± 14.57^a,b,c^	<0.001

PRO = patient-reported outcome; NIH-CPSI = National Institutes of Health Chronic Prostatitis Symptom Index; TCI-R = Temperament and Character Inventory-Revised; PE = premature ejaculation; LPE = lifelong PE; APE = acquired PE; VPE = variable PE; SPE = subjective PE; IELT = intravaginal ejaculatory latency time; NA = not applicable. Data are expressed as mean ± standard deviation. *P^A^*: differences between men in PE and no PE complaint groups were assessed by the dependent *t*-test or chi-square test, as appropriate. *P^B^*: differences among four PE syndromes were assessed by one-way analysis of variance or chi-square test, as appropriate. Differences between two PE syndromes were assessed by *SNK*-q test. ^a^Significant difference compared with LPE. ^b^Significant difference compared with APE. ^c^Significant difference compared with VPE. ^d^Significant difference compared with SPE.

**Table 3:** Correlations between the outcome of PRO, NIH-CPSI, and TCI-R questionnaires in men with PE complaints.

	TCI-R: temperament inventory				TCI-R: character inventory	
	Novelty seeking		Harm avoidance		Self-transcendence	
	Adjusted *r*	*P* value	Adjusted *r*	*P* value	Adjusted *r*	*P* value
PRO questions, scores						
Control over ejaculation	0.35	<0.001				
Satisfaction with sexual intercourse	0.42	<0.001			0.70	<0.001
Severity of PE			0.68	<0.001		
Personal distress	-0.62	<0.001	0.60	<0.001	-0.48	<0.001
Interpersonal difficulty			0.52	<0.001	-0.55	<0.001
NIH-CPSI question, scores						
Total score	-0.55	<0.001	0.60	<0.001	-0.43	<0.001
Pain score	-0.50	<0.001	0.62	<0.001	-0.40	<0.001
Urinary symptoms score			0.66	<0.001		
Quality of life impact score	-0.64	<0.001	0.56	<0.001	-0.69	<0.001

PRO = patient-reported outcome; NIH-CPSI = National Institutes of Health Chronic Prostatitis Symptom Index; PE = premature ejaculation; TCI-R = Temperament and Character Inventory-Revised. Correlations between the outcomes of PRO, NIH-CPSI, and TCI-R questionnaires were analyzed by partial correlations.

**Table 4:** Correlations between the outcomes of NIH-CPSI and TCI-R questionnaires in men with different PE syndromes.

	TCI-R: temperament inventory				TCI-R: character inventory	
	Novelty seeking		Harm avoidance		Self-transcendence	
	Adjusted *r*	*P* value	Adjusted *r*	*P* value	Adjusted *r*	*P* value
Total scores of NIH-CPSI						
LPE	-0.34	<0.001	0.42	<0.001	-0.43	<0.001
APE	-0.70	<0.001	0.68	<0.001	-0.56	<0.001
VPE	-0.44	<0.001	0.36	<0.001	-0.40	<0.001
SPE	-0.52	<0.001	0.40	<0.001	-0.65	<0.001

NIH-CPSI = National Institutes of Health Chronic Prostatitis Symptom Index; PE = premature ejaculation; TCI-R = Temperament and Character Inventory-Revised. Correlations between the outcomes of NIH-CPSI and TCI-R questionnaires were analyzed by partial correlations.

## Data Availability

The data used to support the findings of this study are included within the article.
